# De Garengeot’s hernia patients entirely treated laparoscopically: a safe and feasible alternative—a systematic review

**DOI:** 10.1007/s00423-023-02889-2

**Published:** 2023-05-02

**Authors:** Alberto Gómez-Portilla, Elena Merino, Eduardo López de Heredia, Alberto Gareta, Montserrat Ojeda

**Affiliations:** 1 The University of the Basque Country, EHU/UPV, 01006 Vitoria, Spain; 2 Department of General Surgery, Hospital Santiago Apóstol, University Hospital of Araba (HUA), 01004, Vitoria, Spain

**Keywords:** De Garengeot’s hernia, Femoral hernia appendix, Crural hernia appendix, Laparoscopic hernioplasty, Total extraperitoneal hernia repair (TEP), Transperitoneal preperitoneal hernia repair (TAPP)

## Abstract

**Purpose:**

Less than 450 cases of femoral hernias containing the vermiform appendix have been published since De Garengeot’s first description. A laparoscopic treatment option opened 15 years ago seems reliable and safe. A literature review of all the patients who have benefited from this new therapeutic alternative is presented.

**Methods:**

A systematic review using the German Society of Surgery’s recommendations was performed for De Garengeot’s hernias totally treated laparoscopically. Keywords searched included “De Garengeot hernia” OR “femoral hernia appendix” OR “crural hernia appendix.”

**Results:**

Only 29 out of 225 De Garengeot hernia’s manuscripts were identified describing patients entirely treated laparoscopically: 25 patients by a transabdominal preperitoneal hernia repair (TAPP) and 4 patients by a total extraperitoneal (TEP) procedure; 85.1% were females. The mean age was 71 years. Twenty-two patients had pre-operative imaging tests, sonography (2), computed tomography (14), or both (6). Nevertheless, only 56% had a preoperative diagnosis. Twenty-one cases required urgent treatment, while programmed surgery was possible in 7 instances. An appendix-sparing procedure could be done in 16% of the TAPPs. No postoperative complications occurred. The median hospital stay was 2.5 days.

**Conclusions:**

The best surgical approach for a De Garengeot’s hernia is not defined, and many critical questions remain unanswered. A better understanding of the diagnosis and treatment of this peculiar hernia will supply guidelines for clinicians who may encounter it hereafter. A fully laparoscopic approach seems perfectly safe and feasible for this entity, and it could be considered the first-line alternative if enough expertise is available.

## Background and purpose

A vermiform appendix within a femoral hernia has been known as De Garengeot’s hernia since Rene-Jaques Croissant De Garengeot described this clinical picture in 1731 [1]. It is an infrequent entity, with less than 450 cases published in the literature. This hernia appears almost exclusively on the right side, mainly in females. It debuts practically invariably in an emergency setting as an incarcerated, not reducible, femoral hernia. Acute appendicitis, when present, tends to be the consequence of the strangulation of the appendix by the rigid hernia neck. Clinical signs of peritonitis are usually absent. The preoperative diagnosis of a De Garengeot’s hernia by imaging tests using sonography (US), computed tomography (CT), or magnetic resonance (RMN) remains uncommon. It is generally diagnosed intraoperatively during an emergency repair for an incarcerated hernia. The stated standard treatment procedure for this type of hernia has been classically a simultaneous appendicectomy and primary hernia repair, mainly with an open approach through a groin incision and eventually combined with a mixed laparotomy. The emergence of a minimally invasive approach as a treatment option, opened 15 years ago by Commann et al. [2], seems to be a more reliable method in terms of patient safety because of the simultaneous solution of both pathologies when needed or with the sparing of the appendix when possible. Few patients have benefited from this therapeutic alternative to date. A systematic review of all the manuscripts with De Garengeot’s hernia patients utterly treated by an entirely laparoscopic approach, either by a totally extraperitoneal (TEP) or a transabdominal preperitoneal (TAPP) hernia repair, is presented.

## Methods: literature search strategy

A systematic review was performed, using the methodology of the German Society of Surgery’s recommendations for systematic reviews in general surgery [3], for cases of De Garengeot hernias wholly treated laparoscopically. Search engines utilized included PubMed, Google Scholar, Embase, Ovid, and Web of Science. Keywords searched included “De Garengeot hernia” OR “femoral hernia appendix” OR “crural hernia appendix” OR “femoral appendicitis” OR “crural appendicitis.” The search took place in April 2022.

### Inclusion criteria

English language manuscripts or foreign language papers with an English abstract were included if they described cases with an established diagnosis of a De Garengeot´s hernia. Additionally, all the selected patients reported needed to have been treated entirely laparoscopically. An additional hand-search of all references of the elected manuscripts was done, looking for additional cases not found on the initial database query.

### Article selection

All papers were reviewed for eligibility independently by two reviewers. Discrepancies for inclusion or exclusion were resolved by consensus agreement.

### Statistical analysis

Data from each manuscript were collected by one reviewer and tabulated into a spreadsheet. Quantitative data were analyzed as median and interquartile ranges (IQR), and qualitative data were displayed as frequencies and percentages.

## Results

### Literature review

Four hundred forty-two manuscripts were identified on the initial search for “De Garengeot hernia” OR “femoral hernia appendix” OR “crural hernia appendix” OR “femoral appendicitis” OR “crural appendicitis” (PubMed: 75 + 3 + 1 + 4 + 0, Google Scholar: 107 + 2 + 3 + 2 + 5, Embase: 107 + 2 + 3 + 4 + 5, Web of Science: 107 + 2 + 3 + 2 + 5). After manuscripts were screened, assessed for eligibility, removed duplicates, and references screened for additional reports, 447 patients with a De Garengeot’s hernia with enough information for inclusion were identified in this review. But, finally, only 29 papers [2, 4-31] had De Garengeot’s patients entirely treated laparoscopically and constitute the objective of this systematic review (Table 1). Most patients came from isolated case reports published worldwide in the last 5 years.

**Table 1:** De Garengeot’s hernia patients entirely treated laparoscopically

Author (ref) year	Gender	Age	Imaging test	Preop diagnosis	Surgery	Technique	Appendectomy	Mesh	PO days	PO complications
Comman [2] 2007	F	38	US	No	Urgent	TAPP	Yes	Yes	6	None
Legnani [4] 2007	N.A	65	N.A	N.A	Urgent	TAPP	No	Yes	2	N.A
Ohta [5] 2009	F	72	US + CT	Yes	Urgent	TEP	Yes	Yes	4	None
Matsukawa [6] 2012	F	49	N.A	N.A	Urgent	TEP	Yes	Yes	N.A	None
Beysens [7] 2013	F	64	US + CT	No	Urgent	TEP	Yes	Yes	3	None
Takadate [8] 2014	F	59	CT	Yes	Urgent	TAPP	Yes	Yes	N.A	None
Snoekx [9] 2014	F	76	US + CT	Yes	Elective	TAPP	Yes	No	N.A	None
Saito [10] 2014	F	77	CT	Yes	Urgent	TAPP	Yes	No	N.A	None
Al-Subaie [11] 2015	F	59	NO	No	Elective	TAPP	Yes	Yes	2	None
Nonoyama [12] 2015	M	84	US + CT	Yes	Urgent	TAPP	Yes	N.A	N.A	None
Nonoyama [13] 2016	F	79	N.A	N.A	Urgent	TAPP	Yes	N.A	N.A	None
Díaz [14] 2016	F	64	CT	No	Urgent	TAPP	Yes	Yes	2	N.A
García-Amador [15] 2016	F	77	CT	Yes	Urgent	TAPP	Yes	No	6	None
Yoshida [16] 2017	F	72	N.A	N.A	N.A	TAPP	Yes	Yes	N.A	None
Ikram [17] 2018	F	71	CT	No	Urgent	TAPP	Yes	Yes	2	None
Sinclair [18] 2018	F	81	CT	No	Urgent	TAPP	Yes	No	9	N.A
Anheier [19] 2018	F	68	US + CT	No	Urgent	TAPP	Yes	Yes	3	N.A
Danbara [20] 2019	F	85	CT	Yes	Elective	TAPP	Yes	Yes	N.A	None
Plumbee [21] 2019	M	84	CT	Yes	Urgent	TAPP	Yes	No	3	None
Gómez Portilla [22] 2020	F	83	CT	No	Elective	TAPP	No	Yes	2	None
Abdelwahed [23] 2020	F	75	CT	Yes	Elective	TAPP	Yes	Yes	2	N.A
Sartori [24] 2020	F	63	US	No	Urgent	TAPP	Yes	Yes	3	None
Sakon [25] 2021	F	49	CT	Yes	Urgent	TEP	Yes	Yes	3	None
Fujiwara [26] 2021	M	85	CT	Yes	Urgent	TAPP	Yes	Yes	3	None
Sommerhaldser [27] 2021	F	71	NO	No	Elective	TAPP	No	Yes	2	None
Cohen [28] 2021	M	68	CT	Yes	Urgent	TAPP	Yes	Yes	1	None
Imataki [29] 2021	F	70	CT	Yes	Elective	TAPP	No	Yes	N.A	None
Altiner [30] 2022	F	63	NO	No	Urgent	TAPP	Yes	N.A	1	None
Martín-Arroyo [31] 2022	N.A	83	US + CT	Yes	Urgent	TAPP	Yes	N.A	N.A	N.A

*F* female, *N.A.* not available, *M* male, *US* ultrasound, *CT* computer tomography, *TAPP* transperitoneal preperitoneal approach; total extraperitoneal hernia repair, *TEP* total extraperitoneal

### Patient characteristics

A total of 29 patients with a De Garengeot’s hernia entirely treated laparoscopically were collected with a mean age at presentation of 71 years (IQR 38–85 years). This pathology affects mainly females (85,1%, *n* = 23), always on the right femoral side.

## Diagnosis

Although 22 patients had a pre-operative imaging test, using ultrasound sonography (US) (2) or computed tomography (CT) (14), or both (6), the diagnosis was mostly made at the time of surgery. Only in 14 patients (56%), with enough information available, was a properly preoperative diagnosis of a De Garengeot’s hernia established. The sensitivity of a CT scan to correctly diagnose a De Garengeot hernia was 70% (14 out of 20 true positives), while the sensitivity for the US was only 12.5% (1 out of 8 true positives).

### Operative approach

While 21 cases (72.4%) required urgent treatment, seven patients (24.1%) could be treated on an elective basis. In one patient, no information was available.

One of the patients electively treated underwent a two-stage strategy, initially with prompt drainage of an inguinocrural abscess followed, 8 weeks later, by a delayed TAPP appendectomy plus herniorrhaphy [9]. In a second patient treated with a two-stage procedure, a robotic minimally invasive TAPP hernioplasty was undertaken 4 days after an initial laparoscopic appendectomy [27]. Another case was electively operated on 7 days after the initial diagnosis, treated during the interval with antibiotics “to prevent the potential development of appendicitis” [28].

Twenty-five patients (86.2%) were fully treated by a TAPP approach and four patients (13.8%) by a TEP hernia repair.

A laparoscopic appendectomy was performed in 86.2% of the patients (*n* = 25). Still, in 4 cases, an appendix-sparing hernioplasty could be satisfactorily done, as the appendix was normal after the resolution of the hernia sac, always during TAPP procedures. In all but five patients [9, 10, 15, 18, 21], a prosthetic mesh could be used for the hernia repair. A polypropylene mesh was used most of the time.

### Outcomes

The median hospital stay, reported in 68.9% of cases (*n* = 20), was 2.5 days (IQR 1–9). Moreover, two patients [28, 30] could be discharged the same day, and eight patients the day after. All the patients with specific information available had no postoperative complications. In six cases, there was not enough information available [4, 14, 18, 19, 23, 31].

## Discussion

The patients of this series were typical. Most reported cases included old women with a several-day history of groin swelling just below the right inguinal ligament, inferior and lateral to the pubic tubercle. The node was nonreducible but not strangulated. None of the patients developed signs of peritonitis. The absence of typical symptoms made the preoperative diagnosis of a De Garengeot hernia challenging. Imaging has been used to differentiate and obtain an accurate diagnosis. None of the cases reviewed were successfully diagnosed preoperatively with sonography alone. And, with a sensitivity of 70% for the CT scans available, only 56% patients were properly preoperatively diagnosed. Coronal and sagittal reconstructions have been shown to aid in the reliable identification and classification of these femoral hernias by experienced radiologists.

Most of the cases (72.4% in this series) required emergency surgical treatment. We highlight, once again, the usefulness of laparoscopy as a valuable tool in the diagnosis and treatment of this unusual presentation of femoral hernias.

Commann et al. [2] opened the minimally invasive approach as a treatment option in 2007. With a TAPP procedure they evaluated the intraperitoneal situation, repositioned the hernia contents, made the laparoscopic appendectomy, and repaired the hernia defect.

The laparoscopic TEP approach for a De Garengeot hernia repair was first reported by Otha et al. in 2009 [5]. They initially performed the TEP hernia repair, followed by the laparoscopic appendectomy at once. Instead, Beysens et al. [7] performed first the laparoscopic appendectomy and, later, after the closure of the peritoneal space, the hernia repair using the TEP technique. Both approaches try to overcome the placement of a mesh in a possibly contaminated environment.

Although some advocate removing the appendix in all cases, whether an appendectomy is mandatory in an otherwise non-inflamed appendix is under question. In this series, twenty-five patients (86.2%) suffered an appendectomy, but in three of them (12%), no signs of true appendicitis were present in the final histologic study [7, 10, 17]. In five other patients [4, 22, 27, 29, and our second unpublished case], an appendix-preserving procedure could be guaranteed without further complications.

A mesh repair of the femoral hernia defect was possible in all but four patients [9, 10, 15, 18]. In these contaminated scenarios, the overall low mesh infection rate can likely be attributed to the parietal peritoneum acting as a natural barrier. The peritoneum is closed after the mesh is placed in the preperitoneal space in TAPP and TEP techniques. There is no ulterior contact between the mesh and the abdominal cavity, minimizing the risk of implant contamination.

A totally laparoscopic approach for De Garengeot’s hernias was associated with an overall low complication rate. No complications were reported in these cohort published, and patients had an uneventful recovery and short hospital length of stay. Notably, nine patients were referred with a day-after or less hospital discharge [4, 11, 14, 17, 22, 23, 27, 28, 30], representing a low-invasive treatment strategy.

## Conclusions

The best surgical approach for a De Garengeot’s hernia is not defined nor standardized, and many critical questions remain unanswered. A deeper and better understanding of the diagnosis and treatment of this peculiar hernia will provide guidelines for clinicians who may encounter it in the future. The awareness of the disease and the more frequent use of CT scan imaging may increase the pre-operative diagnosis rate. Undoubtedly, pre-operative diagnosis may affect the urgency of operative intervention and surgical management, leading to improved outcomes.

An entirely simultaneous laparoscopic approach to both pathologies, appendicular and femoral hernia, reinforced mainly by a TAPP approach, seems perfectly safe and feasible to treat this entity. Furthermore, laparoscopy allows an optimal exploration of the whole abdominal cavity and the discovery of possible associated entities. In addition, the period dedicated to delivering and repairing the hernia and its defect represents an acceptable “test time” for the proper assessment of the appendiceal macroscopic viability, allowing a chance to perform an appendix-sparing procedure, avoiding thus unnecessary appendectomies in selected cases, and preventing the contamination of the mesh and the femoral operative field.

When a De Garengeot hernia is clinically suspected, urgent surgical treatment should be performed as soon as possible. A totally laparoscopic approach should be considered the first-line alternative when enough expertise is available, in cases where no necrosis occurs of the appendix is found [29].

## Declarations

All authors declare that the work described has not been published before; that it is not under consideration for publication anywhere else; and that its publication has been approved by all co-authors.
